# Anti-PD-L1 F(ab) Conjugated PEG-PLGA Nanoparticle Enhances Immune Checkpoint Therapy

**DOI:** 10.7150/ntno.65544

**Published:** 2022-01-16

**Authors:** Christina K. Lee, Danielle F. Atibalentja, Lilian E. Yao, Jangho Park, Sibu Kuruvilla, Dean W. Felsher

**Affiliations:** 1 Division of Oncology, Departments of Medicine and Pathology, Stanford University School of Medicine, Stanford, CA, USA; 2 Division of Hematology, Department of Medicine, Stanford University School of Medicine, Stanford, CA, USA

**Keywords:** PD-L1, PEG-PLGA, MC38, Immunotherapy

## Abstract

**Background:** Immune checkpoint therapies are effective in the treatment of a subset of patients in many different cancers. Immunotherapy offers limited efficacy in part because of rapid drug clearance and off-target associated toxicity. PEG-PLGA is a FDA approved, safe, biodegradable polymer with flexible size control. The delivery of immune checkpoint inhibitors such as anti-PD-L1 (α-PD-L1) via PEG-PLGA polymer has the potential to increase bioavailability and reduce immune clearance to enhance clinical efficacy and reduce toxicity.

**Methods:** The Fc truncated F(ab) portion of α-PD-L1 monoclonal antibody (α-PD-L1 mAb) was attached to a PEG-PLGA polymer. α-PD-L1 F(ab)-PEG-PLGA polymers were incubated in oil-in-water emulsion to form a α-PD-L1 F(ab)-PEG-PLGA nanoparticle (α-PD-L1 NP). α-PD-L1 NP was characterized for size, polarity, toxicity and stability. The relative efficacy of α-PD-L1 NP to α-PD-L1 mAb was measured when delivered either intraperitoneally (IP) or intravenously (IV) in a subcutaneous mouse colon cancer model (MC38). Antibody retention was measured using fluorescence imaging. Immune profile in mice was examined by flow cytometry and immunohistochemistry.

**Results:** Engineered α-PD-L1 NP was found to have pharmacological properties that are potentially advantageous compared to α-PD-L1 mAb. The surface charge of α-PD-L1 NP was optimal for both tumor cell uptake and reduced self-aggregation. The modified size of α-PD-L1 NP reduced renal excretion and mononuclear phagocyte uptake, which allowed the NP to be retained in the host system longer. α-PD-L1 NP was non-toxic *in vitro* and *in vivo*. α-PD-L1 NP comparably suppressed MC38 tumor growth. α-PD-L1 NP appeared to elicit an increased immune response as measured by increase in germinal center area in the spleen and in innate immune cell activation in the tumor. Finally, we observed that generally, for both α-PD-L1 NP and α-PD-L1 mAb, the IP route was more effective than IV route for tumor reduction.

**Conclusion:** α-PD-L1 NP is a non-toxic, biocompatible synthetic polymer that can extend α-PD-L1 antibody circulation and reduce renal clearance while retaining anti-cancer activity and potentially enhancing immune activation.

## Introduction

Checkpoint inhibitors that target CTLA-4 and PD-1/PD-L1 have exhibited success in the treatment of both solid and hematological malignancies leading to FDA approval 1-6. However, only about 20-40% of patients show response to immunotherapy 7. Current immunotherapy offers limited benefit due to poor tumor-specific distribution, rapid clearance (half-life of 24 hours to 3 days), and off-target distribution/toxicity 6, 8-12. Approximately 3-40% of injected monoclonal antibodies (mAb) reach the solid tumor tissue 13, 14. This is in part because mAbs are rapidly cleared through glomerular filtration in the kidneys due to their size being less than 10 nm (mAb average size, 5.2-7.1 nm). Fatal immune-related adverse effects range from 15-70% depending on the regimens 15-18. To address these pharmacologic limitations, various nanoparticles have been developed that can potentially improve mAb delivery and retention while reducing toxicity 19.

We investigated whether the checkpoint inhibitor, α-PD-L1 mAb, loaded onto a biodegradable polymer could be used to enhance their pharmacologic properties. Antibodies are composed of an antigen binding fragment F(ab) region and a constant Fc region 20. The Fc portion of mAb results in immune clearance 21-23. This elicits immune-related toxicity such as colitis, hepatitis, pneumonitis 24-27. We reasoned that we may reduce immune clearance and toxicity by loading the F(ab) of α-PD-L1 mAb onto a poly(ethylene glycol)-poly(lactic-co-glycolic acid) (PEG-PLGA) delivery system.

Nanoparticle coating with a water-soluble, “stealth-like” polymer such as PEG has been shown to protect the payload from rapid clearance and enhance the α-PD-L1 mAb biodistribution properties 28, 29. PEG and PLGA are FDA-approved nanoparticle carriers that exhibit versatile functionality, synthetic feasibility, and have already been shown to improve pharmacokinetics, tumor distribution, and safety profile of drug payloads 30-34. Passive targeting is facilitated by the enhanced permeation and the retention (EPR) effect from the leaky vasculature and compromised lymphatic system of the tumor tissue 35. Active targeting is achieved by the display of tumor-specific targeting ligands or antibodies 35. Hence, we inferred that we may enable tumor-specific delivery of the payload through both passive and active targeting to tumor tissue.

We hypothesized that α-PD-L1 NP will exhibit improved therapeutic efficacy as a result of employing the pharmacokinetics of the PEG-PLGA nanoparticles and removing the Fc portion of α-PD-L1 mAb. Our results show that α-PD-L1 NP improves the circulation time of α-PD-L1 mAb via the modification of its pharmacologic properties and maintains the anti-tumor activity in a MC38 colorectal tumor model. This proof-of-concept study establishes that α-PD-L1 F(ab)-conjugated PEG-PLGA nanoparticles may be useful as a platform technology to be investigated for other therapeutic mAbs. We also highlight the importance of characterizing both NP stability and route of delivery.

## Results

### Synthesis and characterization of α-PD-L1 F(ab)-PEG-PLGA nanoparticles

**Figure 1:**
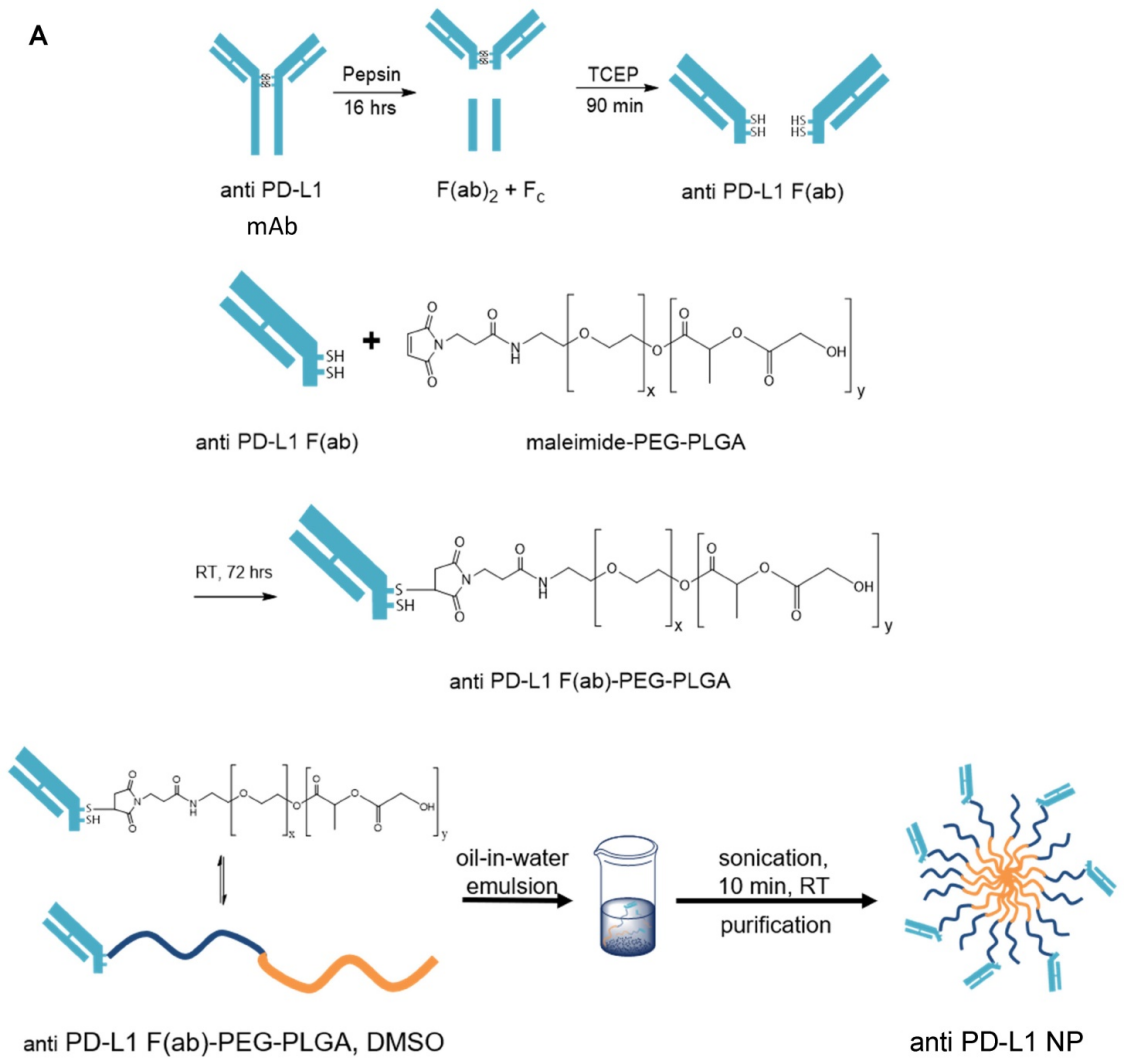

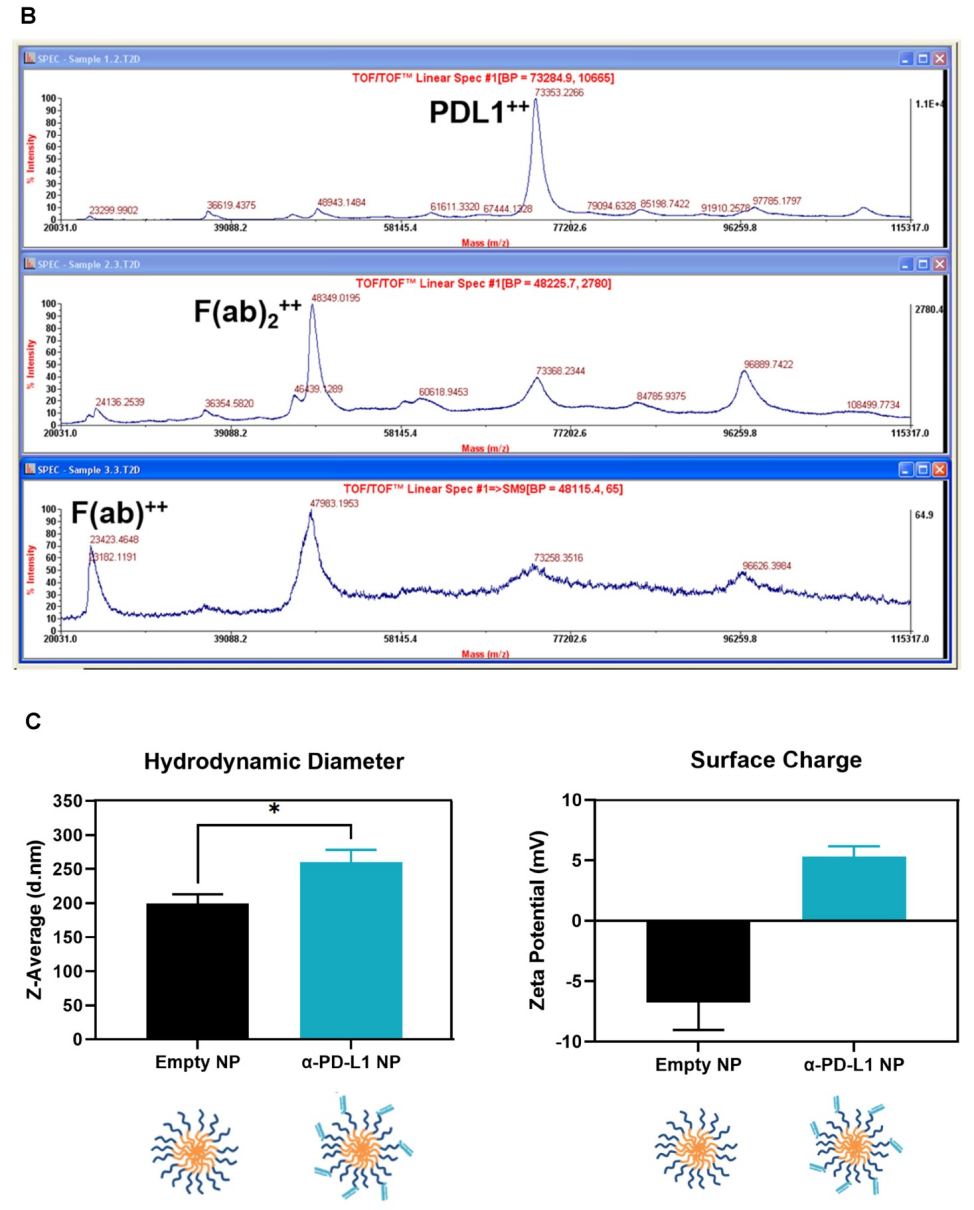
** Nanoparticle characterization.** (A) Schematics of α-PD-L1 F(ab)-PEG-PLGA synthesis. The Fc portion of the antibody is detached to reduce immune clearance and the remaining F(ab) portion is attached to PEG-PLGA to increase nanoparticle size in order to prevent antibody from premature clearance. PLGA is used to control the degradation rates or drug release rates. Water-soluble synthetic polymer, PEG, is used as a protein carrier, to reduce the immunogenicity of the conjugated proteins. (B) MALDI-TOF of α-PD-L1 Fragmentation. MALDI-TOF of α-PD-L1 Fragmentation shows presence of both F(ab) and F(ab)_2_. The presence of F(ab)_2_ is not relevant in the next antibody-to-polymer conjugation step since F(ab)_2_ lacks the free thiol groups exposed on F(ab) that is necessary for conjugation with maleimide linker on PEG-PLGA polymer. The filtration step following the conjugation step eliminates any free antibody fragments that did not bind to polymers. (C) Size and surface charge of the empty NP (n=16) and α-PD-L1 F(ab)-PEG-PLGA (n=27) measured by 90Plus Particle Size Analyzer, Zetasizer Nano ZS90 and 90Plus Zeta Potential Analyzer.

We generated α-PD-L1 F(ab)-PEG-PLGA nanoparticles (α-PD-L1 NP) intended to reduce premature clearance and off-target immune-mediated toxicity (**Figure 1A**). Since the Fc region of the α-PD-L1 mAb is recognized by the Fc receptors on immune compartments leading to off-target immune activation, we first fragmented the α-PD-L1 antibodies into F(ab) and Fc portions as confirmed by the molecular weights measured by MALDI-TOF (**Figure 1B**). We then loaded the F(ab) portion of the α-PD-L1 mAb onto PEG-PLGA to enhance pharmacologic properties. A maleimide linker functionalized to the end of PEG-PLGA polymers was used to conjugate to the free thiol groups exposed on the F(ab) after reduction. The α-PD-L1 NP was formed by using a standard oil-in-water emulsion procedure and the geometry of the nanoparticles was confirmed using Dynamic Light Scattering (DLS) and Zeta Potential measurements (**Figure 1C**). After attachment to PEG-PLGA polymers, the geometry of the antibodies was increased to above the limit for renal excretion of greater than or equal to 10nm (α-PD-L1 NP, average size 260.2nm) with a surface charge that falls between -10mV and 10mV (average peak 5.3mV). Both the size and charge of the nanoparticle fell in ranges that are favorable for cellular uptake and avoid self-aggregation between the nanoparticles 36, 37. The modified size and surface charge are intended to minimize the rapid clearance of the antibodies after intravenous (IV) or intraperitoneal (IP) injection while staying below 500 nm to avoid unintentional uptake by the mononuclear phagocytic system (MPS) 38. Our results indicate that the α-PD-L1 NP are synthetically feasible with amenable pharmacokinetics.

**Figure 2:**
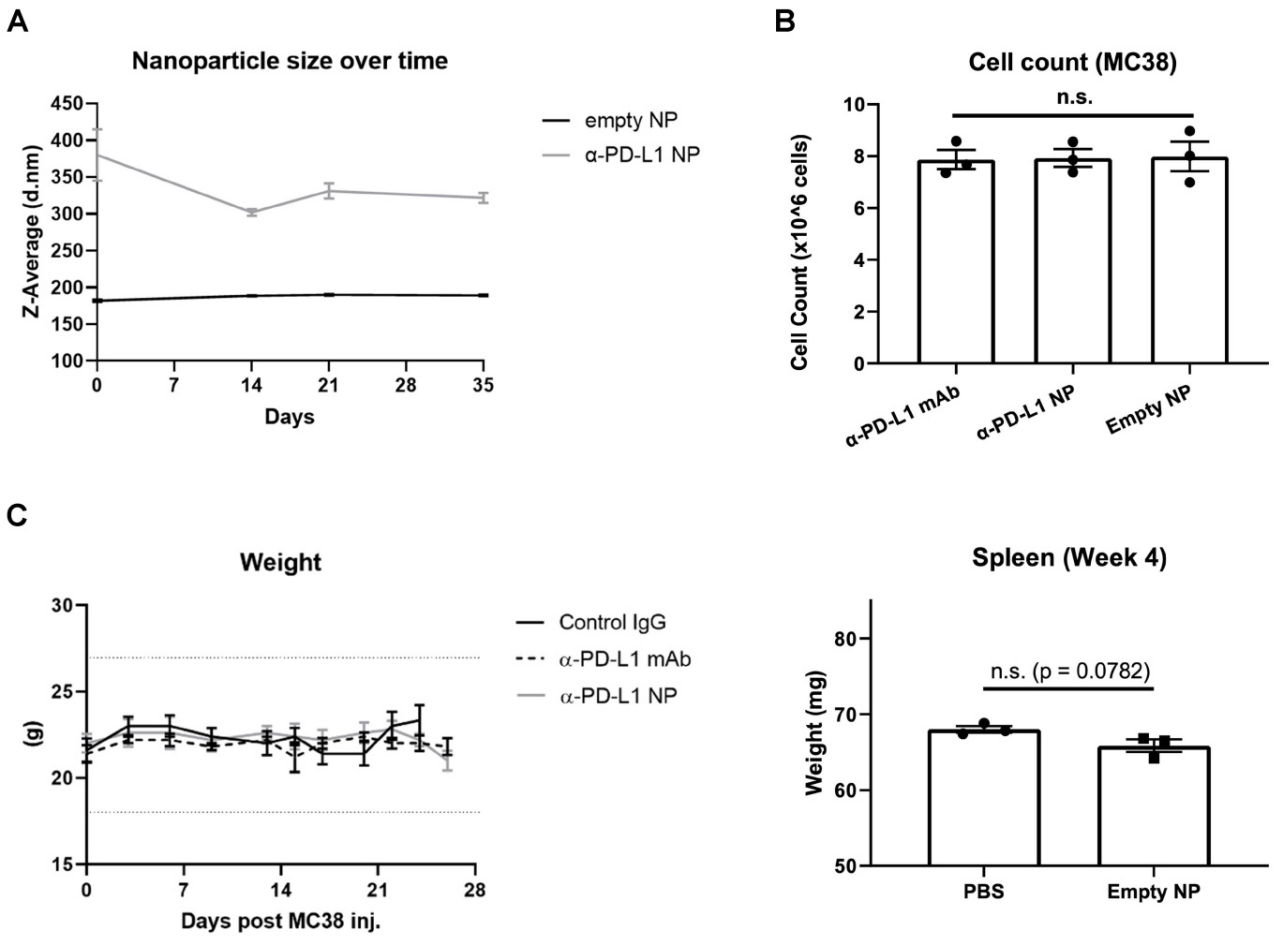
** Nanoparticle Stability and Toxicity.** (A) Size of empty NP and α-PD-L1 NP were tracked over time using Zetasizer Nano ZS90. (n=3 in each group). (B) Cell number of MC38 were counted 26hours after culture with 10µg/ml α-PD-L1 mAb, α-PD-L1 NP, or empty NP. (C) Body weight of healthy mice that received control IgG, α-PD-L1 mAb, or α-PD-L1 NP were recorded every 3-4 days (n=3; 200µg/mice, 3 injections over 9 days;* left*). 20% weight loss is a criterion for euthanasia (*dotted line above and below*). Spleen weight of healthy mice that received either PBS or empty NP was measured at 4 weeks post treatment (n=3; *right*).

We next characterized the stability of α-PD-L1 NP over time. The size of PEG-PLGA (empty NP) and α-PD-L1 NP were measured over 10 weeks with the DLS method. The size of empty NP remained consistent over the period of 10 weeks (PEG-PLGA, average size = 199.4nm). However, the size of α-PD-L1 NP fluctuated by up to 21 percent over 2 weeks and gradually declined to a size comparable to that of empty NP by 10 weeks (**Figure 2A**). We also noted that the size of α-PD-L1 NP varied widely between six batches made at different time points for separate *in vivo* experiments. The size ranged from 147-450.1nm as measured by DLS and transmission electron microscopy (TEM) while the size of the empty NP varied from 142.1nm to 294.55nm (**Figure S1**). This data suggests there is a change in stability of α-PD-L1 NP over time. For all our experiments, we manufactured new nanoparticles and validated their size.

### Toxicity in vitro and in vivo

Next, we evaluated the toxicity of α-PD-L1 NP using *in vitro* and *in vivo* model systems. Murine colon tumor cells, MC38, were cultured in complete media with either α-PD-L1 mAb, empty NP, or α-PD-L1 NP. Total cell number was assessed after two doubling time (doubling time measured appx. 13hours). The cell numbers did not differ significantly between the three groups and the cultures doubled at a consistent rate (**Figure 2B**). As expected, α-PD-L1 mAb had no direct effects on tumor cell viability. We also examined the effects of α-PD-L1 NP *in vivo*. Control mice without tumor exposure were injected intraperitoneally with control IgG, α-PD-L1 mAb, or α-PD-L1 NP. We did not find any significant changes between the treatment groups with regards to mice weight, spleen size, and behavior over the course of two months (**Figure 2C**). We conclude that α-PD-L1 NP has no apparent toxicity *in vitro* or *in vivo.*

### Extended circulation time of immune checkpoint inhibitor

**Figure 3:**
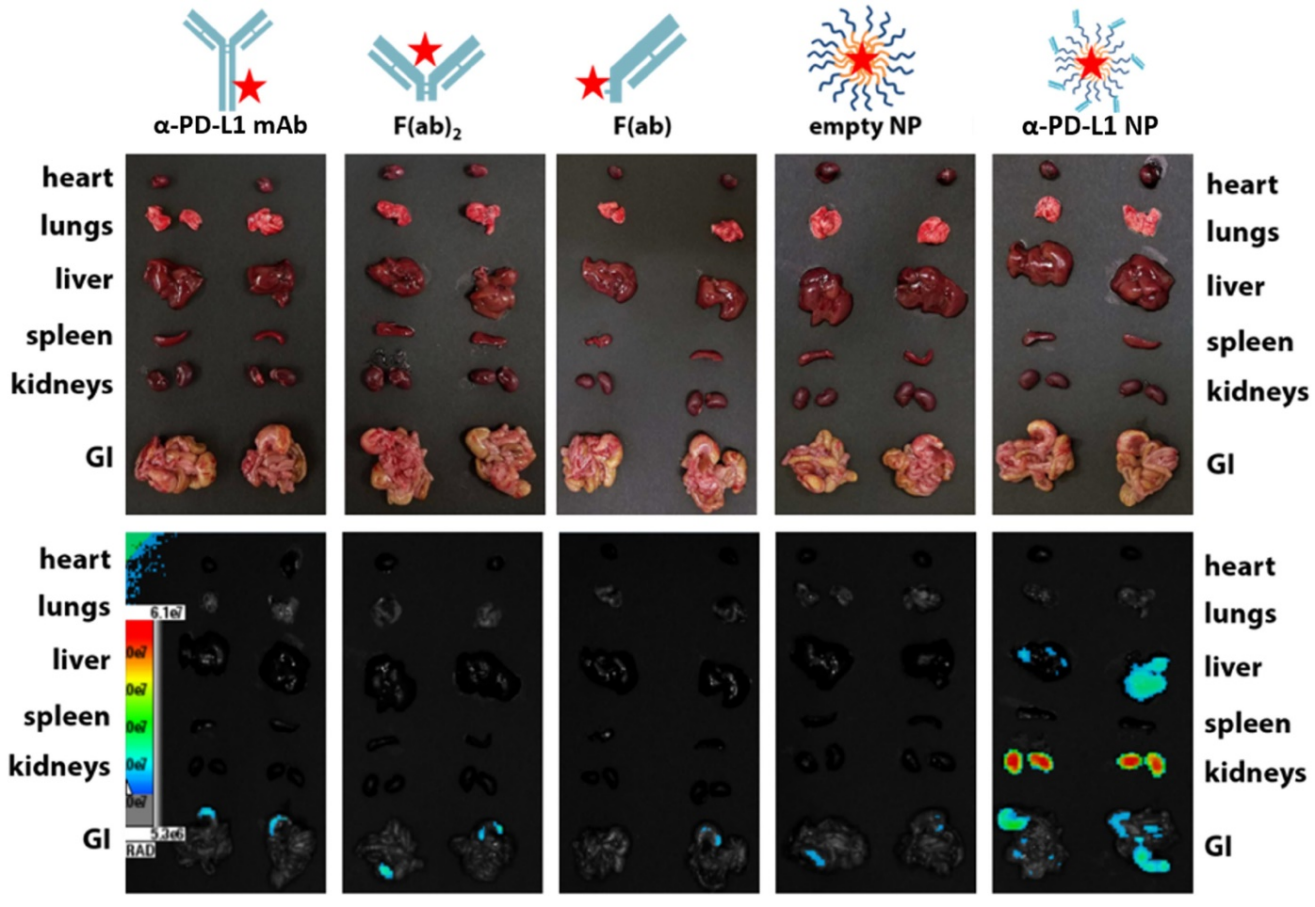
***In vivo* retention of intravenously injected NP compared to α-PD-L1 mAb**. Cy5 dye tagged α-PD-L1 mAb, F(ab)2, F(ab), empty NP, and α-PD-L1 NP were injected into NSG mice IV and fluorescence in different organs was traced 24hours post injection (500 µg of mAb and an equivalent amount of NPs were injected to mice).

We examined the kinetics and distribution of α-PD-L1 NP *in vivo*. A Cy5 dye was used to label α-PD-L1, α-PD-L1 F(ab)_2_, α-PD-L1 F(ab), empty NP, and α-PD-L1 NP and achieved equivalent fluorescent labeling (data not shown). The fluorescently-labeled agents were injected intravenously at equivalent Cy5 concentration into NSG mice, a commonly employed mouse model for fluorescence imaging due to their white coat. Mice were monitored via fluorescence imaging at 0, 4, 8, and 24 hours. The organs of mice that received α-PD-L1 F(ab)-PEG-PLGA had higher expression of fluorescence after 24 hours compared to those that received other regimens. The majority of the fluorescent signal disappeared in the four control groups in the liver, spleen, and kidney while a strong signal was maintained in the α-PD-L1 NP group (**Figure 3**). Thus, the size modification obtained by attaching the F(ab) portion of the immune checkpoint inhibitor to PEG-PLGA allowed the nanoparticles to persist longer in recipient mice.

### Route of drug administration

**Figure 4:**
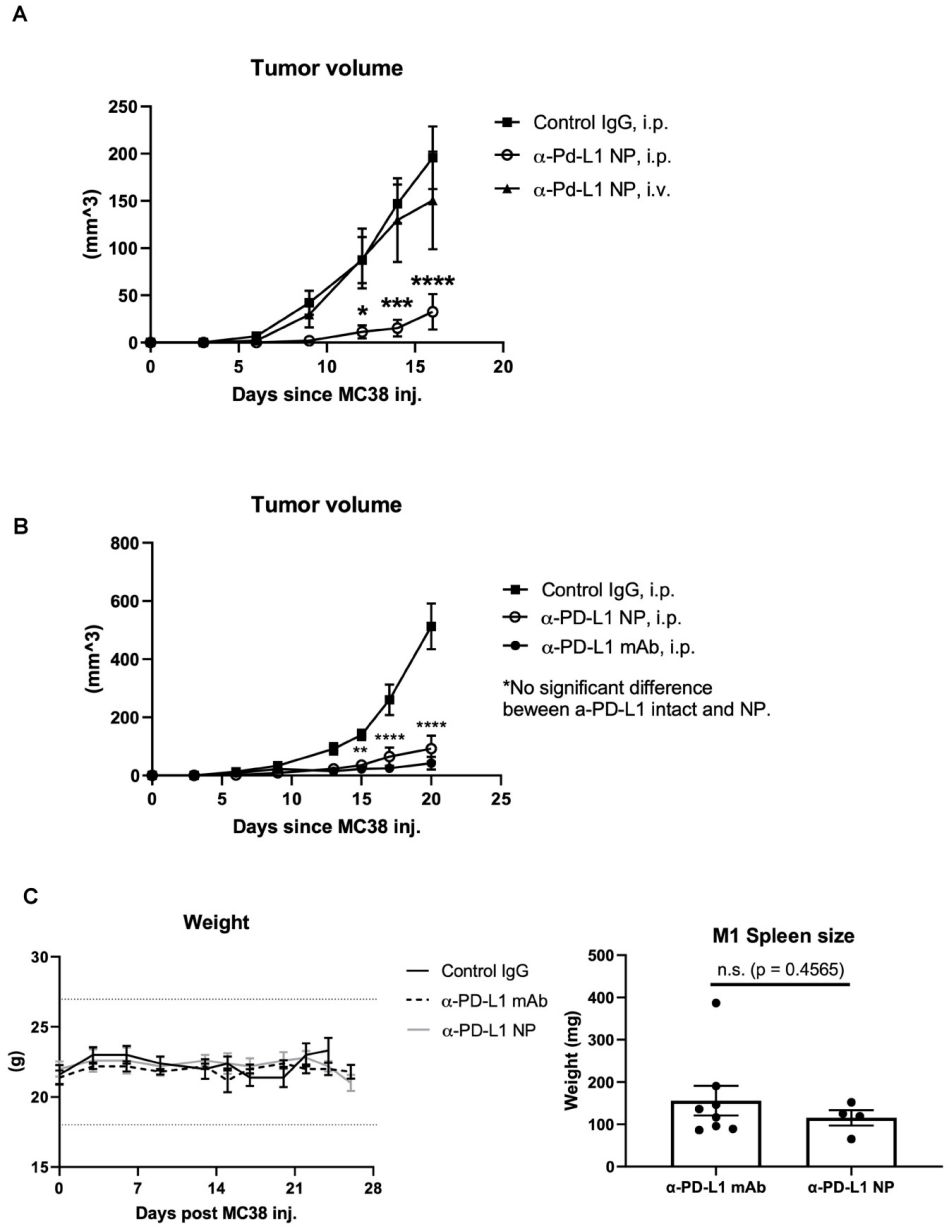
***In vivo* effects of injected NP based on route of administration.** (A) Tumor volumes were measured in mice that received α-PD-L1 NP IP (n=5) versus IV (n=4) until the first mouse reached endpoint as dictated by protocol. Control IgG (n=3) was injected IP as standard practice (200µg/mice, 3 injections over 9 days). (B) Tumor volumes between mice that received control IgG, α-PD-L1 mAb, or α-PD-L1 NP were monitored every 3-4 days post MC38 injection for 3 weeks (n=5 in each group; 200µg/mice, 3 injections over 9 days). (C) Body weight of mice that received control IgG, α-PD-L1 mAb, or α-PD-L1 NP IP were monitored every 3-4 days post MC38 injection for 24-26days (n=5 in each group; *left*). Spleen weight was measured at the time mice were euthanized (*right*).

The route of drug administration can influence therapeutic activity 39, 40. We introduced α-PD-L1 NP either through tail vein (IV) or intraperitoneal (IP) to mice orthotopically transplanted with the MC38 tumor cell line. We found that tumor bearing mice that received α-PD-L1 NP through the IP injection had significantly reduced tumor growth compared to tumor bearing mice that received α-PD-L1 NP IV (**Figure 4A**). Intratumoral (IT) injection of immunotherapies is another route of administration commonly employed in medical research 41. We compared the tumor growth curve in mice treated with α-PD-L1 NP IT, α-PD-L1 NP IP, IgG IP and found that mice treated with α-PD-L1 NP IT and IP exhibited delayed tumor growth compared to mice treated with IgG IP (**Figure S2**). However, there was significant variation within the α-PD-L1 NP IT group, which led us to focus on α-PD-L1 NP IP for subsequent studies. Our findings suggest that IP versus IV injection may improve the efficacy of α-PD-L1 NP.

### Therapeutic efficacy

We compared the therapeutic efficacy of α-PD-L1 NP to α-PD-L1 mAb. MC38 tumors were grown SC in syngeneic C57BL/6J mice which were then treated IP with either α-PD-L1 mAb or α-PD-L1 NP and tumor volume was assessed after 3 weeks. Tumor volumes in mice that received either α-PD-L1 mAb or α-PD-L1 NP were significantly smaller (mean tumor vol. 42.1 mm^2^ and 92.3 mm^2^, respectively) compared to tumor volumes in mice that received the control IgG (mean tumor vol. 513.2 mm^2^). There was no significant difference in tumor size between mice that received α-PD-L1 NP and α-PD-L1 mAb at day 20 post tumor cell injection (**Figure 4B**). We infer that α-PD-L1 NP and α-PD-L1 mAb exhibit similar anti-tumor activity *in vivo*.

### Changes in host immune activation

**Figure 5:**
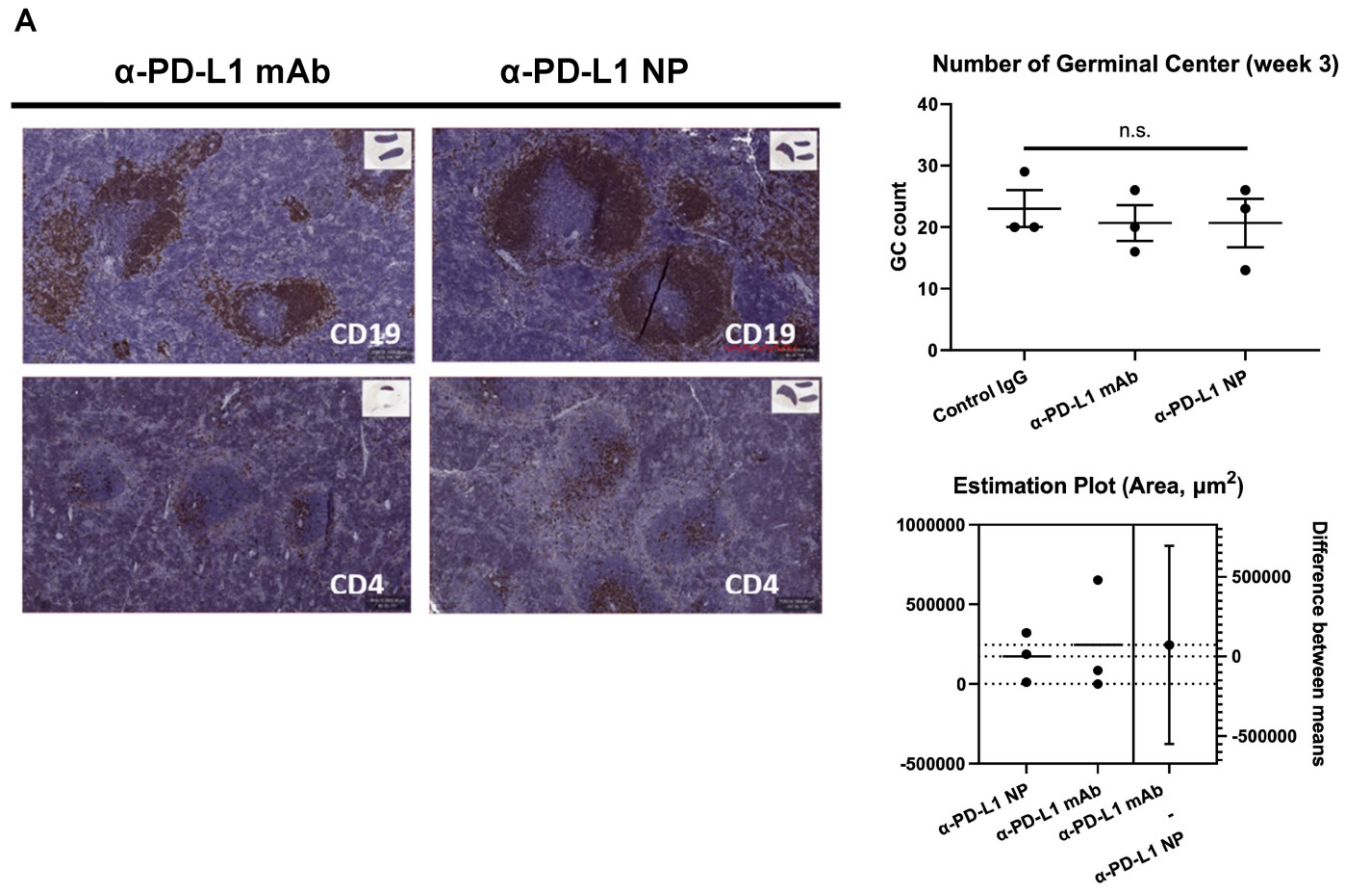

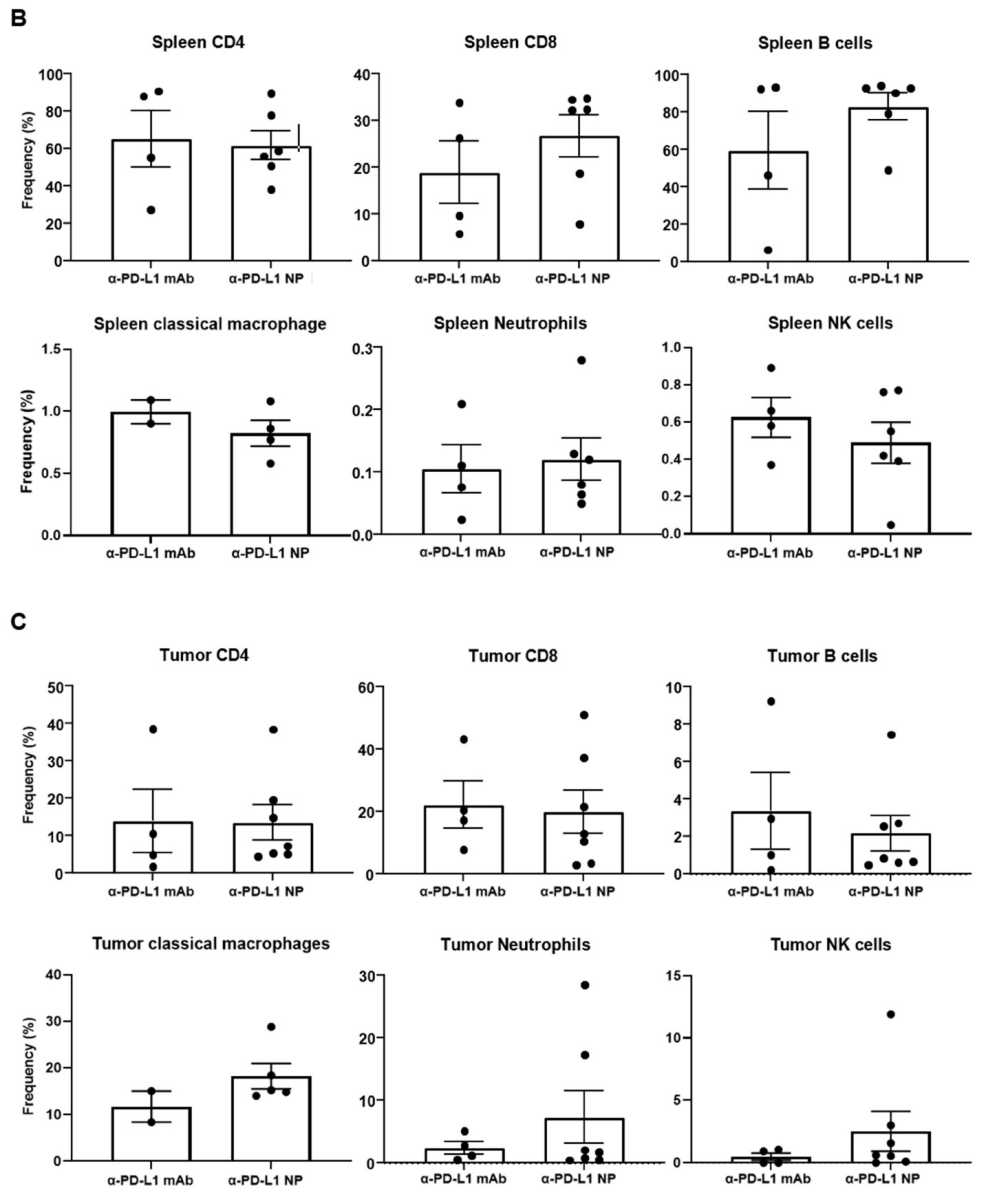
** Change in immune profile over time.** (A) IHC of CD4 and CD19 were used to identify the GC region and quantify the number of GC in the spleen at 3 weeks post MC38 injection (n=3 in each group). Flow cytometry was used for immune profiling of spleen (B) and tumor (C) of mice that received α-PD-L1 mAb or α-PD-L1 NP 4 weeks post MC38 injection.

Blocking PD-L1 on myeloid and lymphoid cells has been found to be important for responses to checkpoint blockade therapies 42-45. We noted that treatment with α-PD-L1 mAb and α-PD-L1 NP both increased spleen size in mice compared to control mice (α-PD-L1 mAb and α-PD-L1 NP, mean spleen weight at 156.1mg and 115.5mg, respectively, **Figure 4C**; healthy mice, mean spleen weight at 68mg, **Figure 2C**). We next measured the number and area of germinal centers (GC) in the spleen associated with activated CD4^+^ T cells and B cells 46-48. Immunohistochemistry staining of CD4 and CD19 was used to estimate the GC number and area in the spleens just before the appearance of splenomegaly at 3 weeks post MC38 injection. The mice that received α-PD-L1 mAb and α-PD-L1 NP exhibited no significant difference in GC number (IgG, 23; α-PD-L1 mAb, 20.7; α-PD-L1 NP, 20.7) and area (α-PD-L1 mAb, 246,235.8µm^2^; α-PD-L1 NP, 173,370.6µm^2^), and had comparable CD4^+^ T cell and B cell numbers at 3 weeks post MC38 injection (**Figure 5A**). Thus, α-PD-L1 NP and α-PD-L1 mAb appear to induce similar immune activation early in the disease course.

We proceeded to examine the immune phenotype of cells in the spleen and tumor using multi-parameter flow cytometry. While not statistically significant, mice that received α-PD-L1 NP versus α-PD-L1 mAb showed an increase in the proportion of CD8^+^ T cells (26.8 versus 18.9 percent) and B cells (83.1 versus 59.5 percent) in the spleen, and a higher frequency of tumor infiltrating MHCII^+^Ly6C/G^+^F4/80^+^ macrophages (18.2 versus 11.7 percent), MHCII^-^Ly6C/G^+^CD11b^+^ neutrophils (7.3 versus 2.4 percent) and NK1.1^+^CD49b^+^ NK cells (2.5 versus 0.5 percent) in the tumor at 4 weeks post MC38 injection (**Figure 5B**). No differences were found in CD4+ T cells in the spleen (α-PD-L1 mAb, 65.4 percent; α-PD-L1 NP, 61.9 percent) or tumor (α-PD-L1 mAb, 13.9 percent; α-PD-L1 NP, 13.5 percent) (**Figure 5C**). Treatment with α-PD-L1 NP demonstrated an immune profile suggestive of immune activation as indicated by higher frequencies of CD8^+^ T cells and B cells in the spleen, and tumor infiltrating inflammatory macrophages, neutrophils, and mature NK cells 49, 50.

## Discussion and Conclusion

Various antibody conjugated nanoparticles are currently being explored to enhance targeted therapies 51. Polymeric NPs are preferred for their easy size modification and biodegradability 52-54. However, immune checkpoint therapies using pegylated nanoparticles have shown limited benefit in overall survival and increased toxicity in patients 55, 56. Stability and drug efficacy of polymeric NP based therapies depend on the carrier type and strategies for antibody conjugation 57, 58.

The work described herein is a proof-of-concept study investigating the feasibility of attaching α-PD-L1 F(ab) fragments onto PEG-PLGA polymers to improve the efficacy of α-PD-L1 therapy. Our study indicates we can generate a nanoparticle with size and surface charge favorable for cellular uptake while avoiding nanoparticle aggregation, premature clearance, and MPS uptake. Further, α-PD-L1 NP significantly increased antibody retention *in vivo* and did not appear to have toxicity. We noted α-PD-L1 F(ab) attached to PEG-PLGA is prone to degradation and clustering 20, 59. Due to its nano-range size, a small change can result in significant size variation between batches and within the same batch over time. We found it was important to characterize α-PD-L1 NP immediately before use to reduce this potential confounding factor. Taken as a whole, our results suggest that PEG-PLGA mediated delivery of α-PD-L1-F(ab) may be a strategy to extend α-PD-L1 antibody retention and reduce toxicity. This method may be useful for other monoclonal antibody therapies.

Particle filtration in spleen, liver, and kidney increases with size. However, the maximal filtration found in the spleen is for particles sized 400 nm and above 60, liver for 107 nm and above 61, and kidney for 10nm and above 62. The average size of our a-PD-L1-PEG-PLGA nanoparticle is 260.2nm. As expected, we found no trace of our engineered NPs in the spleen, a reduced expression in the liver, and the strongest expression in the kidney at 24 hours post injection. The reason why the empty nanoparticle (PEG-PLGA, average 199.4nm) was not similarly identified in the liver and kidney compared to the α-PD-L1 NP is unclear and requires more in-depth analysis. We suspect that the dual function of direct binding to PD-1/PD-L1 as well as slight differences in NP size may introduce different pharmacodynamics.

We found that the route of drug administration is an important factor in α-PD-L1 NP efficacy. Antibodies are commonly injected IV into the host circulatory system to allow rapid target recognition through leaky vasculature in cancer. However, our α-PD-L1 NP exhibited significantly improved efficacy when delivered IP compared to IV. This may be because α-PD-L1 NP that is introduced directly into systemic circulation risks being entrapped in the liver and lung 63-65. In addition, α-PD-L1 NP that is injected IP must pass through the lymphatic system before entering the circulatory system. α-PD-L1 NP that travels through the lymphatic system can interact and educate immune cells in the lymphatic system before tumor site entry 66, 67. Directly injecting α-PD-L1 NP to tumor (IT) had an effect but the mass of tumor may have been too small for the therapy to have a more meaningful result. Additional optimization different from our established treatment model may yield more consistent efficacy. Overall, the delivery of α-PD-L1 NP IP versus IV may be more efficacious.

We found α-PD-L1 mAb and α-PD-L1 NP were associated with similar immune activation. α-PD-L1 NP had higher frequencies of CD8^+^ T cells and B cells in the spleen and higher frequencies of inflammatory macrophages, neutrophils, and NK cells in the tumor. These changes have previously been shown to be favorable in the clinical setting 68, 69. However, we did not see evidence for enhanced efficacy in mice treated with α-PD-L1 NP. This may be because the immune activation observed may not be sufficient to confer increased tumor regression. Alternatively, the MC38 tumor assay we used was not sensitive enough to detect changes in efficacy when compared to the α-PD-L1 mAb. More in-depth evaluation of the impact of tumor heterogeneity and the effect of immunotherapy on tumor microenvironment may also yield a better understanding of the phenomenon we observed. Regardless, we conclude that α-PD-L1 NP demonstrated a comparable immune phenotype to the monoclonal α-PD-L1 mAb.

The discovery of α-PD-L1 therapy has been an important milestone in cancer therapy. However, existing therapies have limitations in efficacy and are associated with toxicity 70, 71. The α-PD-L1 NP approach used in this study may be a platform to improve the therapeutic potential and reduce the toxicity of α-PD-L1 as well as other monoclonal antibody therapies.

## Material and Methods

### Synthesis and Characterization of PD-L1 F(ab) fragments

Anti-mouse PD-L1 (clone 10F.9G2, BioXCell) antibody was fragmented using established protocols. Briefly, the whole IgG was digested with pepsin (ThermoFisher) according to manufacturer's instruction with minor modifications (16 hour incubation). The F(ab)_2_ fragment was collected by purifying the digest using a 50 kDa MWCO centrifuge filter (Vivaspin 500), and then further digested into F(ab) fragments with free sulfhydryl groups after incubation with TCEP HCl (20 mM) for 90 minutes. The F(ab) fragments were purified using 10K MWCO centrifuge filters. MALDI-TOF analysis confirmed the successful fragmentation of the antibody.

### α-PD-L1 F(ab)-PEG-PLGA Conjugation and Formation of Nanoparticles

F(ab) fragments were coupled to MAL-PEG(5k)-PLGA(5k) polymers (Nanosoft Polymers) prepared at 10 mg/mL in 0.1M NaPO_4_, 0.15M NaCl, and 10 mM EDTA solution by incubating at a 1:10 F(ab) to PEG-PLGA ratio. The coupling was run at 4^o^C overnight before purification using 10K MWCO centrifuge filters. To form nanoparticles, α-PD-L1 F(ab)-PEG-PLGA polymers were solvent-replaced with DMSO, and then added dropwise into ultra-pure water at 1:10 v/v ratio. The solution was then homogenized for 5 minutes to form α-PD-L1 F(ab)-PEG-PLGA nanoparticles. The nanoparticles were purified and concentrated using 10K MWCO centrifuge filters.

### Characterization of α-PD-L1 F(ab)-PEG-PLGA Nanoparticles

The particle size of α-PD-L1 NP was measured using Dynamic Light Scattering (DLS) method on a 90Plus Particle Size Analyzer (Brookhaven Instruments, Holtsville, NY) and Zetasizer Nano ZS90 (Malvern Panalytical). For stability experiments, data was collected at day 0, 7, 14, 21, 28, 35, and 70. Raw distribution data was plotted in GraphPad Prism software and fit using a Gaussian curve, with the mean being taken as the particle size for that replicate. The average of three separate replicates was taken to find the mean particle size ± standard error of the mean (SEM). We also determined the zeta potential of the particles using a 90Plus Zeta Potential Analyzer (Brookhaven Instruments, Holtsville, NY) and Zetasizer Nano ZS90. Particle formulations were dissolved in DI water at 1:10 v/v. The average of three separate replicates was taken to find the mean zeta potential ± SEM. The presence of equivalent amount of a-PD-L1 was verified through Nanodrop2000 and injected to mice in *in vivo* study.

### Cell Culture

MC38 cells (generously donated by the laboratory of Dr. Ronald Levy) were cultured in T-75 flasks using DMEM supplemented with 10% FBS, 100 units/ml penicillin/streptomycin, 2mM L-glutamine, and 0.1mM non-essential amino acids. The cells were maintained at 37°C, 5% CO2, and 95% relative humidity. Cells were passaged at 80-90% confluency using a 0.25% trypsin/0.20% EDTA solution. The cell doubling time was established to be 13 hours based on a series of time course cell counts.

### Animal Work

Animals were housed in a pathogen-free environment at Stanford University and all procedures were performed in accordance with Stanford's Administrative Panel on Laboratory Animal Care (APLAC) protocols. For toxicity experiments and tumor development and therapy experiments, age and gender matched C57BL/6J mice (8-12 weeks) were utilized. For biodistribution experiments, NOD scid gamma (NSG) mice (4-6 weeks) were utilized.

### Toxicity

For *in vitro* experiments, MC38 cells were cultured with 10µg/ml of either α-PD-L1 mAb, α-PD-L1 NP, or empty NP and collected for cell count after approximately two doubling cycles. For *in vivo* experiments, 200µg of either control IgG, α-PD-L1 mAb, or α-PD-L1 NP were given IP to healthy mice every 3 days for a total of 3 injections. Body weight and overall condition were recorded for 3-4 weeks. Spleens from healthy mice that received either PBS or empty NP were collected at 4 weeks and 8 weeks for examination of any unintended autoimmune toxicity from PEG-PLGA polymers.

### Biodistribution

To measure the distribution of α-PD-L1 NP, NSG mice were administered with either 500 µg of α-PD-L1 mAb, an equivalent amount of α-PD-L1 NP, or other controls (F(ab)_2_, F(ab), and empty NP) fluorescently-labeled with Cy5 dye intravenously and imaged using an IVIS Spectrum fluorescence imager (Perkin Elmer) to trace the geographical distribution of the treatments over time. Fluorescence was measured at times 0, 4, 8, and 24 hours, and organs were excised to measure fluorescence after 24 hours. This was confirmed after excising vital organs and measuring the fluorescence - the liver, kidneys, and GI all exhibit fluorescent intensity in the α-PD-L1 NP group versus all other control groups.

### Immunohistochemistry staining

Spleens were harvested from age and gender matched C57BL/6 mice at three weeks post MC38 injection and fixed in 10% formalin w/v (4% formaldehyde solution) for 48 hours at room temperature. The tissues were then stored in 70% ethanol until processing for paraffin embedding and sectioning. CD19 (1:800 in 5% skim milk; Cell Signaling, #90176T), CD4 (1:1000 in 5% skim milk; abcam, #ab183685), PD-L1 (1:300 in 1x TBS; Proteintech, #17952-1-AP) were used in IHC according to manufacturer's protocol.

### Flow cytometry

Spleens and tumors were harvested from C57BL/6 mice at 4 weeks following orthotopic transplantation with the MC38 colon adenocarcinoma cell line. Spleens were minced with fine scissors prior to passage through a 70μM nylon mesh filter (fisher scientific, 22363548) and washed with RPMI media (Corning, 21-040-CM). Red blood cells were lysed with ACK lysis buffer (Gibco, A10492-01) prior to resuspension in 1X PBS (Corning, 21-040-CV). Tumors were digested and processed according to manufacturer protocol using a Miltenyi tumor dissociation kit (130-096-730). Tumor cells were agitated after vigorous vortexing at RT in a 37C water bath for 30-60 min prior to washing with RPMI and straining through a mesh filter. Spleen and tumor cells were resuspended in 1ml 1X PBS at 0.5-1 million cells and stained using a Live/Dead fixable near-IR dead cell stain kit fixable at 1:1000 dilution (Invitrogen, L34975). Cells were subsequently washed 2x with PBS, resuspended in 100 μl FACS buffer (PBS supplemented with 2% FBS and 0.5 mM EDTA) and transferred to a U-bottom microtiter plate for staining. Fc block (BD Pharmingen, 553132) was added at 1:50 dilution and cells were incubated for 20 min on Ice followed by addition of 50μl of the prepared antibody cocktail (supplementary table 1). Cells were incubated with antibodies for 30 min on ice, washed twice with 200μl FACS buffer. The cells were resuspended in 100μl 1x fixation/permeabilization buffer ( BD Pharmingen) for 30 min on ice in the dark, washed 2x with 1X permeabilization buffer ( BD Pharmingen) prior to resuspension in 200µl FACS buffer for flow cytometry analysis. Invitrogen UltraComp beads (01-2222-42) were prepared per manufacturer instructions and used to set up compensation. See Supplementary Table 1 for additional information.

### Tumor Development and Therapy

C57BL/6 mice were injected subcutaneously with 0.5x10^6^ or 0.1x10^6^ MC38 cells in 100µl PBS in their right flank and observed every day for palpable tumors. Animals were monitored every 3 days once tumor mass was detected. Body weight, tumor size, and overall condition were recorded. Animals were randomly enrolled into study groups (control IgG, α-PD-L1 mAb, or α-PD-L1 NP) and treatment begun 3-7 days based on the number of MC38 cells injected. 200µg of control IgG, α-PD-L1 mAb, or α-PD-L1 NP was injected in final volume of 100µl either IP or IV (based on the study objective) every 3 days for total of 3 injections. Palpable tumors were measured using calipers. Tumor volume was calculated V = π/6 x (length) x (width) x (height). When any of the tumor dimension reached ≥17mm or mice lost more than 20% of their body weight, they were euthanized accordingly to protocol. Plasma, spleen, and tumor were collected for immunohistochemistry and flow cytometry.

### Statistical Analysis

Results (mean ± SEM) were analyzed for statistical significance by Student's t-test and one-way ANOVA using GraphPad Prism (Graphpad Software, Inc.). Significance is denoted by * for p<0.05, ** for p<0.01, and *** for p<0.001.

## Supplementary Material

Supplementary figures and table.
