# Supplementary material for: Anti-PD-L1 F(ab) Conjugated PEG-PLGA Nanoparticle Enhances Immune Checkpoint Therapy
Source: Nanotheranostics. 2022 Jan 16;6(3):243–55. doi: 10.7150/ntno.65544 (PMC8824669; doi:10.7150/ntno.65544)
Supplement: Supplementary file 1 — Supplementary figures and table. [file ntnov06p0243s1.pdf]

Supplementary Figure 1: Nanoparticle Size distribution from different Batches.

A

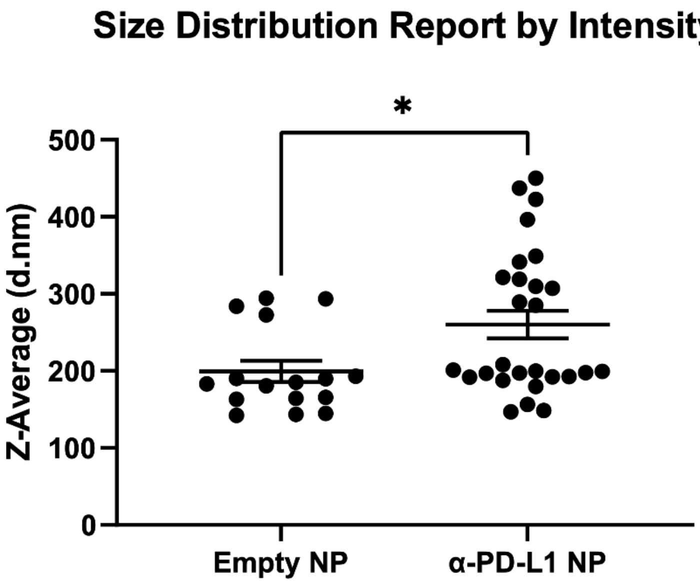

B

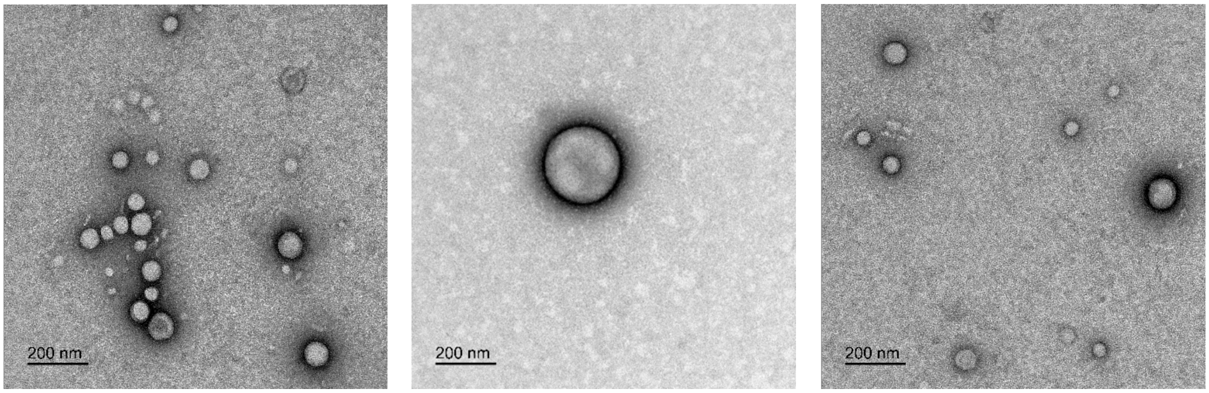

(A) Empty NP and α-PD-L1 F(ab)-PEG-PLGA were measured each time the nanoparticles were generated for experiment. Each dot is representative of individual batches. (B) TEM of α-PD-L1 F(ab)-PEG-PLGA. The scale bar represents 200 nm for all panels.

Supplementary Figure 2: Intratumoral (IT) injection.

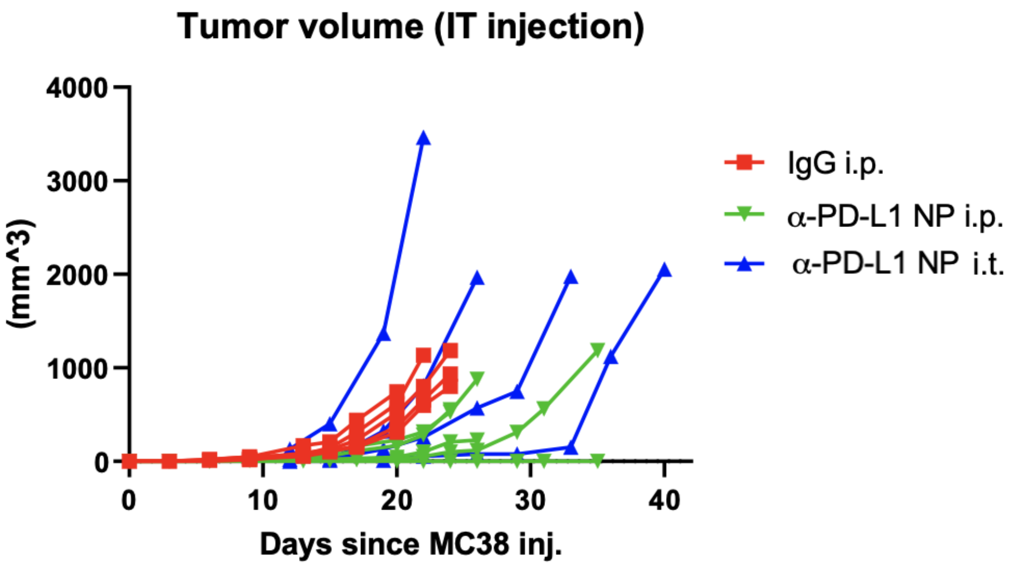

Tumor growth curve *in vivo* when α-PD-L1 NP was injected IP versus IT (mice injected day 4, 7, and 10 post-MC38).

Supplementary Figure 3: PD-L1 expression in tumor.

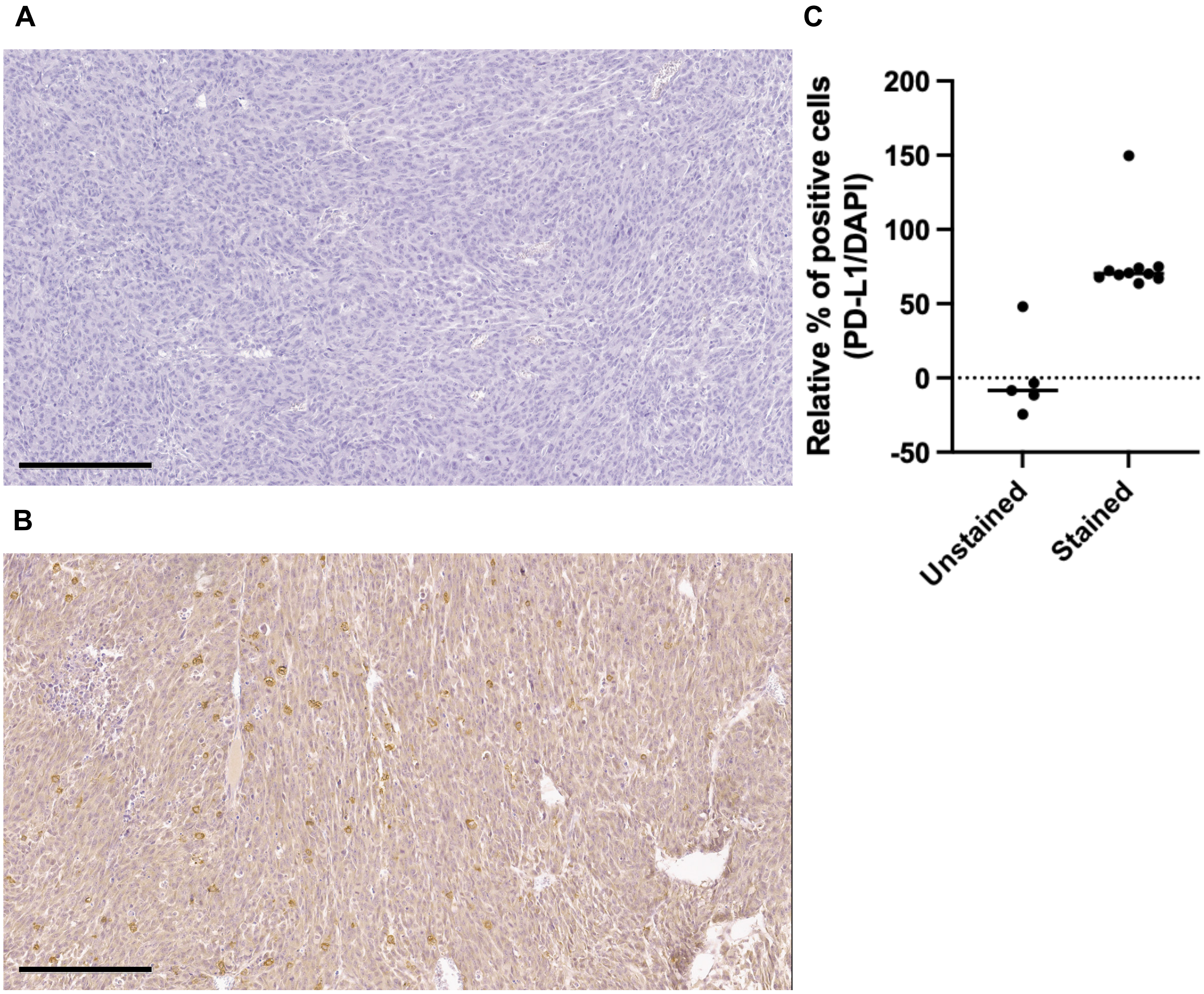

Representative IHC of PD-L1 of tumor at day 24 post MC38 injection. (A) Unstained, (B) stained, and (C) quantification (n=5 mice in IgG IP group). The scale bar represents 200 um.

Supplementary Table 1: Multiparameter Flow Cytometry Antibody List.

Cellular Marker	Fluor	Clone	Manufacturer	Catalog #
CD4	Alexa 488	GK1.5	ebiosciences	53-0041-82
CD8a	PERCP-eFluor710	53-6.7	invitrogen	46-0081-82
CD8a	PECY5.5	53-6.7	ebiosciences	35-0081-82
CD3e	BUV 395	145-2C11	BD Horizon	565992
CD11c	BV 605	HL3	BD Horizon	563057
CD11b	BV786/785	M1/70	Biolegend	101243
NK1.1	BV 650	PK136	BD Horizon	564143
CD19	BV711	1D3	BD Horizon	563157
MHCII	BV 421	M5/114.15.2	Biolegend	107631
B220	Alexa 647	RA3-6B2	ebiosciences	17-0452-82
Ly6C/G	Alexa 700	RB6-8C5	invitrogen	56-5931-82
CD49b	PE	DX5	ebiosciences	12-5971-82
CD49b	PECY5	DX5	ebiosciences	15-5971-80
CD45	PE	30-F11	BD Pharmingen	553081
F4/80	PECY7	BM8	invitrogen	25-4801-82
